# Indoleamine 2,3-dioxygenase 1 deficiency attenuates CCl_4_-induced fibrosis through Th17 cells down-regulation and tryptophan 2,3-dioxygenase compensation

**DOI:** 10.18632/oncotarget.17119

**Published:** 2017-04-15

**Authors:** Weichao Zhong, Lei Gao, Zhenting Zhou, Haiyan Lin, Chun Chen, Peng Huang, Weiliang Huang, Chuying Zhou, Shaohui Huang, Linghui Nie, Ye Liu, Youming Chen, Daqiao Zhou, Zhiping Lv

**Affiliations:** ^1^ School of Traditional Chinese Medicine, Southern Medical University, Guangzhou, Guangdong, 510515, China; ^2^ Department of Liver Diseases, Shenzhen Traditional Chinese Medicine Hospital, Shenzhen, Guangdong, 518033, China; ^3^ Department of Pharmacology, Shantou University Medical College, Shantou, Guangdong, 515041, China; ^4^ The Third Affiliated Hospital of Southern Medical University, Guangzhou, Guangdong, 510630, China

**Keywords:** indoleamine 2,3-dioxygenase 1, liver fibrosis, T helper 17 cells, tryptophan 2,3-dioxygenase, 1-methyl-D-tryptophan

## Abstract

Indoleamine 2,3-dioxygenase 1 (IDO1) is an intracellular rate-limiting enzyme in the metabolism of tryptophan along the kynurenine pathway, subsequently mediating the immune response; however, the role of IDO1 in liver fibrosis and cirrhosis is still unclear. In this study, we investigated the role of IDO1 in the development of hepatic fibrosis and cirrhosis. Patients with hepatitis B virus-induced cirrhosis and healthy volunteers were enrolled. For animals, carbon tetrachloride (CCl_4_) was used to establish liver fibrosis in wild-type and IDO1 knockout mice. Additionally, an IDO1 inhibitor (1-methyl-_D_-tryptophan) was administered to WT fibrosis mice. Liver lesions were positively correlated with serum IDO1 levels in both the clinical subjects and hepatic fibrosis mice. A positive correlation between serum IDO1 levels and liver stiffness values was found in the cirrhosis patients. Notably, IDO1 knockout mice were protected from CCl_4_-induced liver fibrosis, as reflected by unchanged serum alanine transaminase and aspartate transaminase levels and lower collagen deposition, α-smooth muscle actin expression and apoptotic cell death rates. On the other hand, tryptophan 2,3-dioxygenase (TDO), another systemic tryptophan metabolism enzyme, exhibited a compensatory increase as a result of IDO1 deficiency. Moreover, hepatic interleukin-17a, a characteristic cytokine of T helper 17 (Th17) cells, and downstream cytokines’ mRNA levels showed lower expression in the IDO1^–/–^ model mice. IDO1 appears to be a potential hallmark of liver lesions, and its deficiency protects mice from CCl_4_-induced fibrosis mediated by Th17 cells down-regulation and TDO compensation.

## INTRODUCTION

Liver fibrosis is a wound-healing process elicited by various toxic injuries, including viruses, alcohol and fat accumulation, and it is the final common pathway of almost all chronic liver diseases [1]. Liver fibrosis can progress to cirrhosis, which is estimated to affect 1–2% of the world's population [2]. Its complications are major causes of morbidity and mortality worldwide. Despite considerable improvement in our understanding of the fibrogenic process in recent years, the treatment options remain limited [3]. Given that liver fibrosis has become a common health care problem, exploring its underlying mechanisms is of great importance.

Liver fibrosis mainly results from liver injury-mediated inflammation and activation of hepatic stellate cells (HSCs). HSCs can transform into myofibroblasts, secreting large amounts of collagen and synthesizing extracellular matrix (ECM) to facilitate an imbalance in ECM formation and degradation, eventually leading to liver fibrosis [4]. Recent evidence indicates that activation of the immune system is closely related to liver fibrosis [5]. T cell-mediated immune injury plays an important role in the development of chronic liver disease, which is induced by viruses, parasites and chemicals. Generally, in patients with chronic liver disease, CD4+ T cells decrease, while CD8+ T cells increase. CD8+ T cells attack injured hepatocytes infected with pathogens but simultaneously increase the liver damage [6, 7]. Moreover, T helper 17 (Th17) cells, which originate from CD4+ T cells, are a recently discovered subset of T helper cells. Interleukin 17a (IL-17a), the characteristic cytokine of Th17 cells, is a known hepatic fibrosis inducer that mediates ECM remodelling [8–10].

Indoleamine 2,3-dioxygenase (IDO) is an intracellular enzyme that catalyses the first and rate-limiting step in the metabolism of the essential amino acid tryptophan (TRP) in the kynurenine (KYN) pathway [11]. Two types of IDO are known: IDO1 and IDO2 [12]. In both humans and mice, IDO1 is widely distributed but IDO2 expression is limited [13]. The regulation of IDO1 expression is mediated by several immune and inflammatory factors; it is mainly up-regulated by interferon-γ (IFN-γ) and down-regulated by interleukin-4 (IL-4) [14]. Recent studies suggest that IDO1 plays a crucial role in the induction of immune tolerance to tumours. The current study demonstrates that induction of IDO1 and elevation of L-KYN might play a critical role in both the early and late phases of liver carcinogenesis [15]. A previous study revealed that the proliferation of CD8+ and CD4+ T cells is suppressed in response to the TRP-depleted microenvironment caused by IDO1 expression, but the effect is stronger for CD8+ T cells than CD4+ T cells [16]. Furthermore, the proliferation of Th17 cells can be suppressed by general control nonderepressible 2 (GCN2) kinase, which is activated by TRP depletion [17].

In the present study, we investigated the role of IDO1 in a clinical trial and in a carbon tetrachloride (CCl_4_)-induced hepatic fibrosis mouse model to obtain preclinical data for the treatment and/or chemoprevention of liver fibrosis (and even cirrhosis) by modulating IDO1 expression.

## RESULTS

### The serum level of IDO1 was decreased in patients with HBV-induced cirrhosis and positively correlated with the degree of cirrhosis

**Figure 1:**
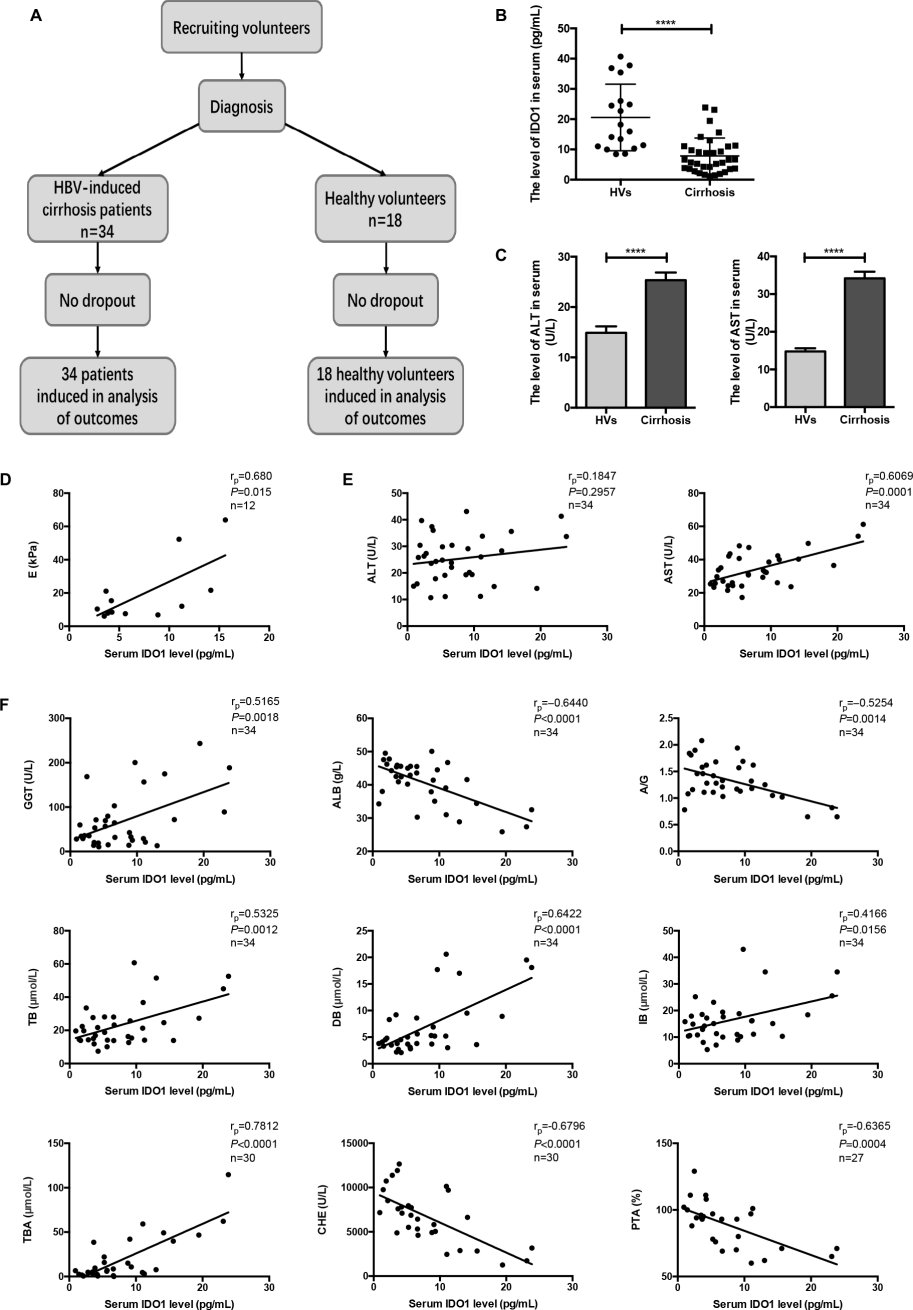
The serum level of IDO1 was decreased in patients with HBV-induced cirrhosis and positively correlated with the degree of cirrhosis (**A**) Participant flow diagram illustrating the two study groups. (**B**) ELISA evaluating the serum IDO1 level in participants. (**C**) The serum levels of ALT and AST in participants were detected with an automatic biochemical analyser. (**D**) Pearson's linear correlation tests for IDO1 and liver stiffness value in patients. (**E**) and (**F**) Pearson's linear correlation tests for IDO1, ALT, AST, GGT, ALB, DB, TBA, CHE and PTA levels in patients’ serum. r_p_: Pearson's correlation coefficient. The data are presented as the means ± SEM (*P < 0.05, **P < 0.01, ***P < 0.001, ****P < 0.0001).

We first conducted clinical trials on patients with hepatitis B virus (HBV)-induced cirrhosis to investigate whether the serum level of IDO1 would change compared with healthy volunteers (HVs) (Figure 1A). The background characteristics of the participants are presented in Table 1. Twelve patients underwent a FibroScan^®^ examination with ECHOSENS^™^, from which we obtained liver stiffness (E) values. Interestingly, the serum level of IDO1 was decreased significantly in the patients with cirrhosis compared with the HVs (Figure 1B). The serum levels of biochemical markers of liver injury, alanine transaminase (ALT) and aspartate transaminase (AST), were significantly higher in the patients than the HVs (Figure 1C). However, liver function was within normal limits in most of the patients. Using Pearson's linear correlation tests, we found a positive correlation between serum IDO1 and E values (Figure 1D). Although there was no positive or negative correlation between serum IDO1 and ALT levels, we found a positive correlation between serum IDO1 and AST levels in the samples from patients (Figure 1E). Moreover, the serum levels of γ-glutamyl transferase (GGT), albumin (ALB), albumin/globulin (A/G) ratio, total bilirubin (TB), direct bilirubin (DB), indirect bilirubin (IB), total bile acid (TBA), cholinesterase (CHE) and prothrombin time activity (PTA) were measured in the samples from patients. The above levels can reflect the degree of hepatocyte damage in liver diseases in various ways, including protein metabolism, bilirubin metabolism and bile acid metabolism. With Pearson's linear correlation tests, we found that the serum IDO1 level was positively correlated with serum GGT, DB and TBA levels (Figure 1F). In addition, the serum IDO1 level was negatively correlated with serum ALB, CHE and PTA levels (Figure 1F). Furthermore, the serum IDO1 level was positively correlated with serum TB and IB, and was negatively correlated with serum A/G ratio (Supplementary Figure 1). Collectively, the results of this clinical study indicated that the serum IDO1 level was decreased in patients with HBV-induced cirrhosis compared with HVs. Moreover, under the condition of cirrhosis, this clinical study indicated positive correlations of the serum IDO1 level with liver lesions and the degree of hepatic fibrosis, despite the reduction in IDO1.

**Table 1:** Background characteristics of subjects

characters	HBV-induced cirrhosis	HVs
NO.	34	18
Gender, male/female	23/11	12/6
Age (years)	49.82 ± 9.44	50.61 ± 7.04
Alanine transaminase (ALT), U/L	25.34 ± 8.94	14.89 ± 5.41
Aspartate transaminase (AST), U/L	34.19 ± 10.25	14.78 ± 3.59
Albumin (ALB), g/L	40.59 ± 6.63	43.78 ± 9.26
A/G ratio	1.33 ± 0.36	1.86 ± 0.25
Total bilirubin (TB), μmol/L	23.31 ± 12.84	14.06 ± 4.32
Direct bilirubin (DB), μmol/L	6.87 ± 5.35	5.18 ± 1.36
Indirect bilirubin (IB), μmol/L	16.44 ± 8.27	8.87 ± 3.01
γ-glutamyl transferase (GGT), U/L	67.74 ± 10.73	ND
Total bile acid (TBA), μmol/L	19.66 ± 4.78	ND
Cholinesterase (CHE), U/L	6683 ± 3061	ND
Prothrombin time activity (PTA), %	89.04 ± 17.10	ND

Values are expressed as mean ± standard deviation.

A/G, albumin/globulin.

ND not determined.

### Serum IDO1 was time-dependently elevated in a CCl_4_-induced fibrosis mouse model and positively correlated with liver lesions

**Figure 2:**
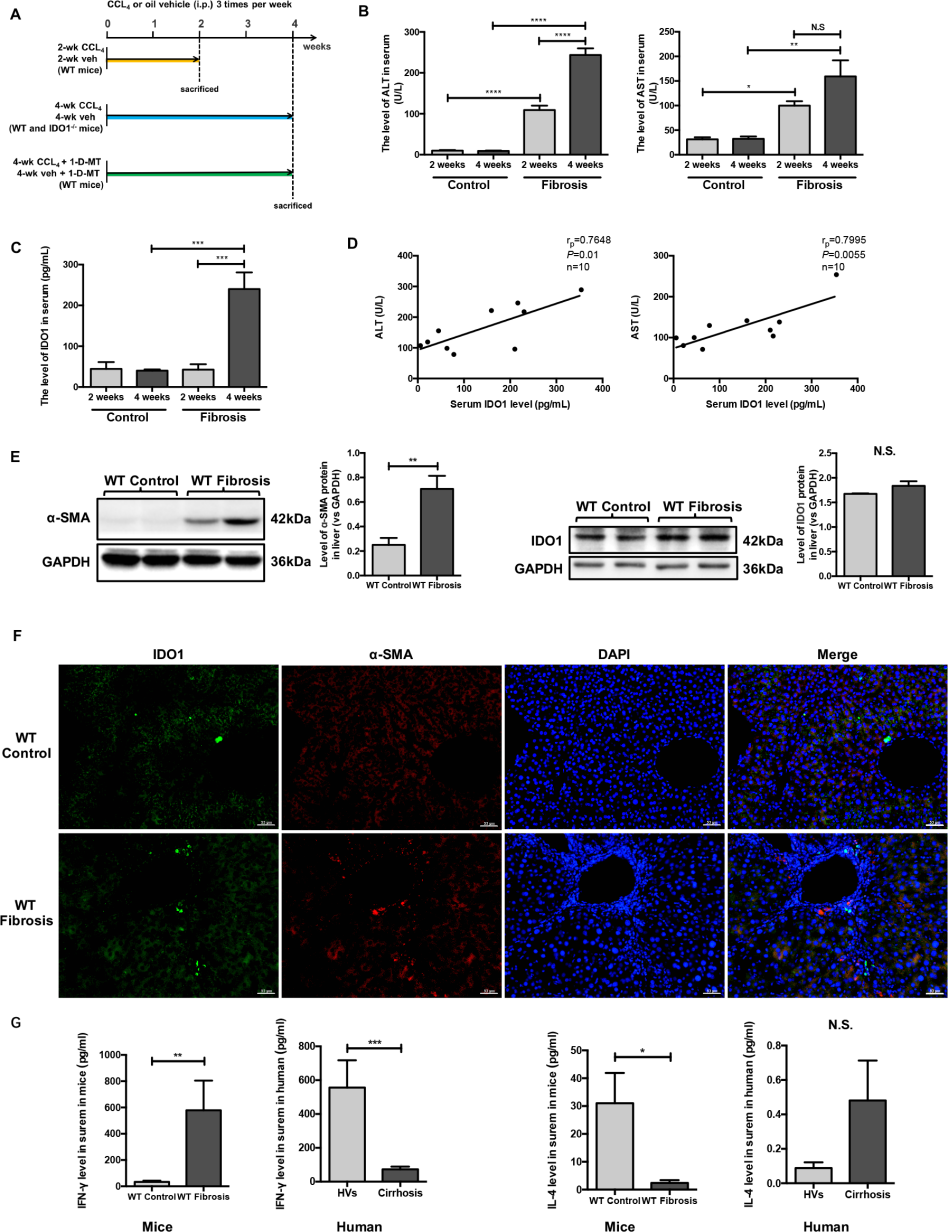
Serum IDO1 was time-dependently elevated in a CCl_4_-induced fibrosis mouse model and positively correlated with liver lesions (**A**) Experimental plan for mice. (**B**) Liver lesions were assessed by measuring serum ALT and AST levels in WT control and model mice. (**C**) ELISA for detecting serum IDO1 level in WT control and model mice. (**D**) Pearson's linear correlation tests for serum IDO1, ALT and AST levels in the WT model mice. (**E**) Western blot analysis of the expression of IDO1 and α-SMA in the livers of WT control and model mice (4-week). (**F**) Immunofluorescence analysis to detect the expression of IDO1 and α-SMA in the livers of WT control and model mice (4-week). (**G**) ELISA for detecting serum IFN –γ and IL-4 in mice and clinical subjects. The data are presented as the means ± SEM (*P < 0.05, **P < 0.01, ***P < 0.001, ****P < 0.0001).

**Figure 3:**
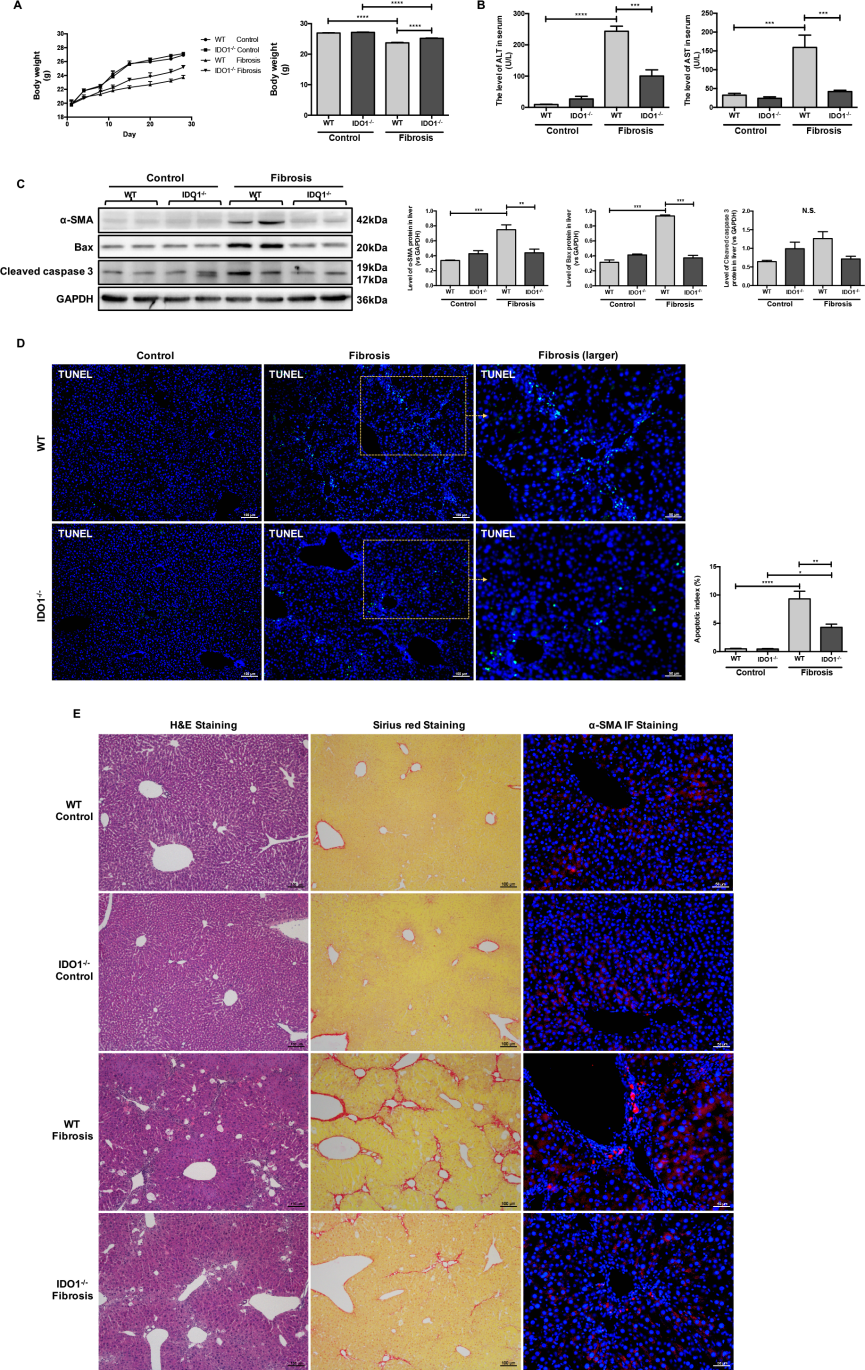
IDO1 deficiency attenuated the development of CCl_4_-induced fibrosis in mice (**A**) Changes in mean body weight in WT and IDO1^–/–^ mice during injection and the body weights of WT and IDO1^–/–^ mice at the end of the 4th week. (**B**) Liver lesions were assessed by measuring serum ALT and AST levels in WT and IDO1^–/–^mice. (**C**) Western blot analysis of the expression of α-SMA, BAX and cleaved caspase 3 in WT and IDO1^–/–^ mice. (**D**) TUNEL assay to detect apoptotic cell death in liver tissues. (**E**) H&E, Sirius red and α-SMA immunofluorescence staining of liver sections. The data are presented as the means ± SEM (*P < 0.05, **P < 0.01, ***P < 0.001, ****P < 0.0).

To evaluate the changes in IDO1 levels in mice, we established a model of liver fibrosis induced by CCl_4_. The mice were sacrificed at the end of 2 weeks or 4 weeks (Figure 2A). As expected, CCl_4_ injury resulted in macroscopically evident liver fibrosis. Among the 4-week mice with liver fibrosis, injection of CCl_4_ induced increases in α-smooth muscle actin (α-SMA) protein, collagen fibers, and α-SMA-positive cells in the liver (Figure 2E, 2F; Figure 3E). Moreover, the expression of IDO1 in the liver of mice with liver fibrosis was increased but was not significantly different from that in the control mice (Figure 2E, 2F). Injection of mice with CCl_4_ induced higher levels of ALT and AST than in control mice. Furthermore, the level of ALT in the 4-week fibrosis mice was increased significantly compared with the 2-week fibrosis mice (Figure 2B). Interestingly, the serum level of IDO1 in the 2-week fibrosis mice remained unchanged compared with the control mice. In contrast with the change in serum IDO1 in human subjects, the level of serum IDO1 increased significantly in 4-week fibrosis mice compared with the control mice (Figure 2C). Pearson's linear correlation tests revealed positive correlations between serum IDO1 and liver lesions (AST and ALT) (Figure 2D), consistent with the results of the clinical study. As mentioned above, IDO1 is mainly up-regulated by IFN-γ but down-regulated by IL-4. Interestingly, the level of serum IFN-γ was significantly increased in 4-week fibrosis mice but was remarkably decreased in the patients with cirrhosis, compared with their control groups respectively. For the serum level of IL-4, it was significantly decreased in 4-week fibrosis mice but showed an up-trend in cirrhosis patients (Figure 2G). These results showed the reason for a reverse trend of the serum level of IDO1 in fibrosis mice and patients with cirrhosis. Furthermore, these findings indicated that in mice with hepatic fibrosis, the level of serum IDO1 remained the same in the state of mild liver lesions but was increased under the condition of severe liver lesions.

### IDO1 deficiency attenuated the development of CCl_4_-induced fibrosis in mice

We then used wide-type (WT) and IDO knocked-out (IDO^–/–^) mice at the age of 6–8 weeks with matched body weights and investigated the role of IDO1 in regulating CCl_4_-induced liver fibrosis (Figure 2A). The body weights of the WT model mice were lower than those of the IDO1^–/–^ fibrosis mice at each time point. The mean body weight of the IDO1^–/–^ mice was significantly higher than that of the WT mice at the end of treatment with CCl_4_ (Figure 3A). Compared with the WT model mice, livers from the IDO1^–/–^ model mice had less severe fibrosis. A histologic examination of liver sections stained with haematoxylin-eosin (H&E) and Sirius red revealed less severe fibrosis in the IDO1^–/–^ mice. Consistently, the IDO1^–/–^ model mice had lower α-SMA expression and fewer α-SMA-positive cells in their liver tissues than the WT model mice (Figure 3E). Furthermore, the levels of serum ALT and AST in the IDO1^–/–^ fibrosis mice were both significantly lower than those in the WT fibrosis mice (Figure 3B), which indicated that hepatocytes were less damaged in the IDO1^–/–^ model mice. The IDO1^–/–^ mice had lower expression of Bcl-2 associated X protein (BAX) and cleaved caspase 3 in liver tissues than WT mice (Figure 3C). A terminal deoxynucleotidyl transferase dUTP nick-end labelling (TUNEL) assessment revealed that the IDO1^–/–^ fibrosis mice had lower rates of apoptotic cell death in liver tissues than the WT fibrosis mice (Figure 3D). Consistently, Sirius red staining revealed less severe fibrosis in the 1-Methyl-_D_-tryptophan (1-_D_-MT) mice (Supplementary Figure 2A). Moreover, the levels of serum ALT and AST in the 1-_D_-WT fibrosis mice were both significantly lower than in the WT fibrosis mice (Supplementary Figure 2B). Moreover, the 1-_D_-MT fibrosis mice had lower expression of BAX and cleaved caspase 3 in liver tissues than did the WT fibrosis mice (Supplementary Figure 2C). These data provided multiple lines of evidence that IDO1 deficiency attenuated the progression of CCl_4_-induced fibrosis in mice.

### IDO1 deficiency elevated the expressions of TDO and GCN2 kinase during liver fibrosis

**Figure 4:**
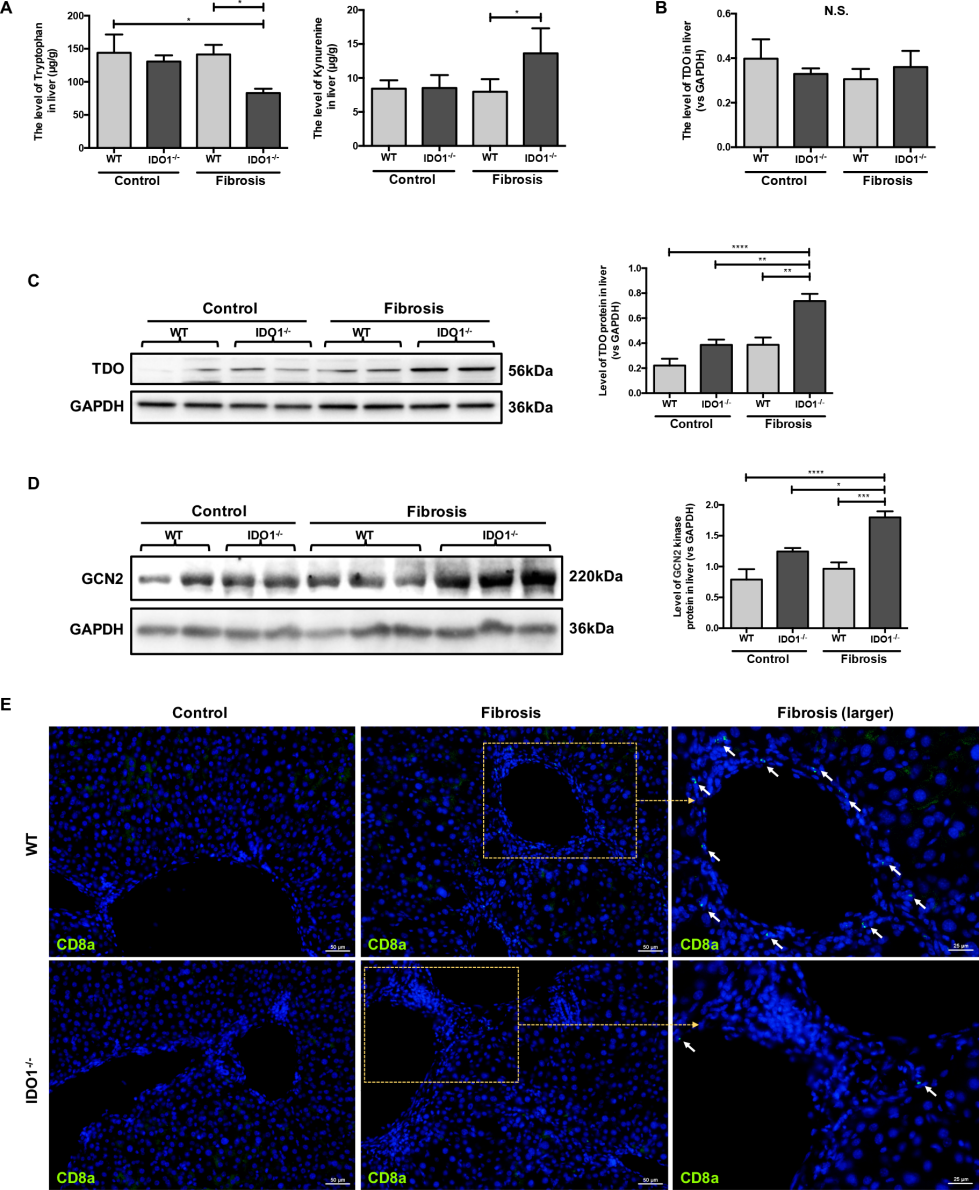
IDO1 deficiency elevated the expressions of TDO and GCN2 kinase during liver fibrosis (**A**) LC-MS/MS analysis to detect the concentrations of TRP and KYN in liver tissues of WT and IDO1^–/–^ mice. (**B**) Quantitative real-time PCR analysis to detect TDO mRNA in the liver tissues of WT and IDO1^–/–^ mice. (**C**) and (**D**) Western blot analysis of the expression of TDO and GCN2 kinase in the livers of WT and IDO1^–/–^ mice. (**E**) Immunofluorescence analysis to detect the expression of CD8a in the livers of WT and IDO1^–/–^ mice. The data are presented as the means ± SEM (*P < 0.05, **P < 0.01, ***P < 0.001, ****P < 0.0001).

We then detected the level of TRP and KYN, which can be mediated by IDO1, in liver tissues in mice. The level of TRP in the IDO1^–/–^ fibrosis mice was significantly lower than those in the WT fibrosis mice. In contrast, the IDO1^–/–^ fibrosis mice had higher level of KYN in liver tissues than did the WT fibrosis mice (Figure 4A). However, the changes of TRP and KYN in IDO1^–/–^ fibrosis mice are contrary to theory. Tryptophan 2,3-dioxygenase (TDO) is another TRP metabolism enzyme in animals [18]. Thus, we further investigated changes in TDO and the effects of TRP metabolism changes on hepatic fibrosis mice. Among the IDO1^–/–^ model mice, the expression of TDO was significantly increased in the liver tissue compared with the WT model mice, although the expression level of TDO mRNA in the liver was comparable between the IDO1^–/–^ model mice and WT model mice (Figure 4B, 4C). After treatment with CCl_4_, the IDO1^–/–^ model mice had dramatically higher expression of GCN2 kinase in liver tissue than did the WT model mice, which was possibility activated by TRP depletion (Figure 4D). Moreover, the expression of CD8a in liver tissue was remarkably decreased in the IDO1^–/–^ model mice compared with the WT model mice (Figure 4E), which could reflect that the expression of GCN2 kinase was elevated indeed. These findings indicated that TDO and GCN2 kinase were overexpressed in hepatic fibrosis liver under the condition of IDO1 deficiency.

### IDO1 deficiency reduced IL-17a and downstream cytokines during liver fibrosis

**Figure 5:**
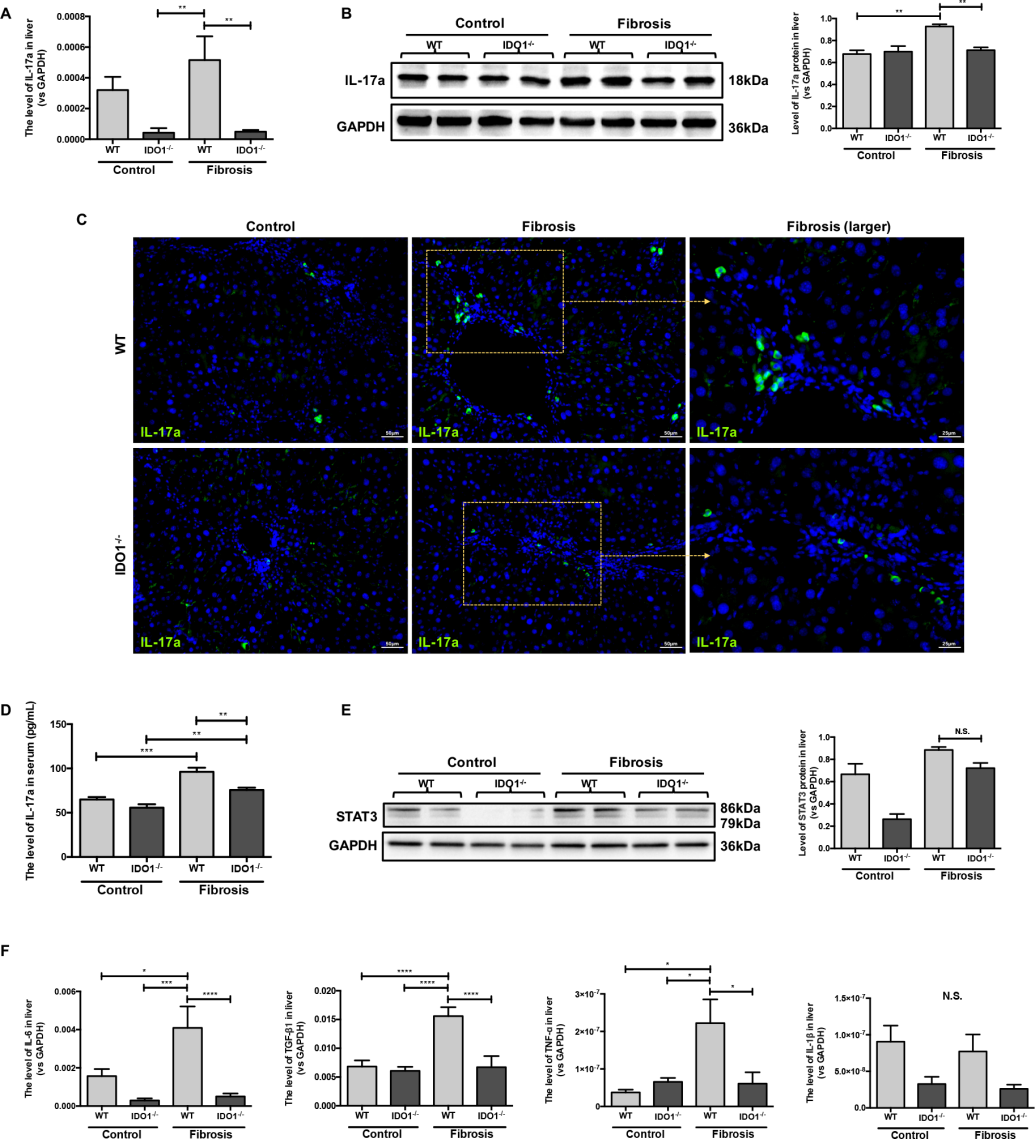
IDO1 deficiency reduced IL-17a and downstream cytokines during Liver fibrosis (**A**) Quantitative real-time PCR analysis to detect IL-17a mRNA in the liver tissues of WT and IDO1^–/–^ mice. (**B**) Western blot analysis of the expression of IL-17a in the livers of WT and IDO1^–/–^ mice. (**C**) Immunofluorescence analysis to detect the expression of IL-17a in the livers of WT and IDO1^–/–^ mice. (**D**) ELISA evaluating the serum IL-17a level in mice. (**E**) Western blot analysis of the expression of STAT3 in the livers of WT and IDO1^–/–^ mice. (**F**) Quantitative real-time PCR analysis to detect IL-6, TGF-β1, TNF-α and IL-1β mRNA in the liver tissues of WT and IDO1^–/–^ mice. The data are presented as the means ± SEM (*P < 0.05, **P < 0.01, ***P < 0.001, ****P < 0.0001).

We further analysed the changes of IL-17a and hepatic mRNA expression levels of interleukin-6 (IL-6), interleukin-1β (IL-1β), tumour necrosis factor-α (TNF-α) and transforming growth factor-β1 (TGF-β1) in mice. The mRNA expression levels of IL-17a were significantly decreased in the IDO1^–/–^ model mice compared with the WT model mice (Figure 5A). Moreover, the IDO1^–/–^ mice had lower expression of IL-17a in liver tissues than WT mice (Figure 5B, 5C). Consistently, the level of serum IL-17a increased significantly in the IDO1^–/–^ model mice compared with the WT model mice (Figure 5D). In addition, the downstream cytokines’ mRNA expression levels of IL-6, TNF-α and TGF-β1 were all dramatically decreased in the IDO1^–/–^ model mice compared with the WT model mice (Figure 5F). Despite the downward trend of IL-1β mRNA levels in the livers of the IDO1^–/–^ model mice, there was no significant difference between the IDO1^–/–^ and WT fibrosis mice (Figure 5F). Furthermore, the expression of signal transducer and activator of transcription 3 (STAT3) protein in liver tissue did not show a significant difference in the IDO1^–/–^ model mice compared with the WT fibrosis mice (Figure 5E). These results reflected that the proliferation of Th17 cells was decreased in liver tissues in IDO1^–/–^ model mice after treatment with CCl_4_, as were the mRNA expression levels of IL-6, TNF-α and TGF-β1.

## DISCUSSION

In the present study, we first discovered that the level of serum IDO1 was significantly decreased in patients with cirrhosis compared to HVs. We then found a positive correlation between the serum level of IDO1 and the degree of liver stiffness in the cirrhosis patients. Moreover, the serum IDO1 level was positively correlated with serum AST, GGT, TB, IB, DB and TBA levels. Additionally, serum IDO1 was negatively correlated with serum ALB, A/G ratio, CHE and PTA levels. Interestingly, the level of serum IDO1 was time-dependently elevated, remaining unchanged in 2-week fibrosis mice but increased in 4-week fibrosis mice compared with the respective control mice. This discrepancy is likely due to changes in the serum levels of IFN- γ and IL-4. Consistent with the results of the clinical study, the level of serum IDO1 was positively correlated with serum ALT and AST in mice. Thus, the level of serum IDO1 might be regarded as a potential hallmark of liver lesions and the degree of liver fibrosis.

As noted above, IDO1 is the rate-limiting enzyme in the degradation of TRP, which exists in the placenta, epididymis, anterior chamber and gastrointestinal tract in addition to the liver [19, 20]. It was recently reported that IDO1 acts as an effector and an indicator of protective immune responses in patients with acute hepatitis B. The activation of IDO1 is vigorous in the early phase and is a hallmark of successful HBV clearance in patients with acute hepatitis [21]. With respect to hepatitis C virus (HCV)-infected liver disease, HCV infection stimulates IDO1 expression in hepatocytes [22]. Moreover, the activation of IDO1 is enhanced in patients with chronic hepatitis C [23, 24]. Consistently, in a diethylnitrosamine-induced liver carcinogenesis mouse model, IDO1 up-regulation contributed to the development of liver carcinogenesis [15]. It was confirmed that in different types of liver disease, the expression or activation of IDO1 changed, which could also affect the progression of disease through its regulation of immune responses. IDO1 can participate in the regulation of the immune response in three ways. First, in the microenvironment, IDO1 can deplete TRP, which is an essential amino acid for bacteria. Thus, IDO1 plays a role in the anti-inflammatory response [25]. More importantly, under the condition of tryptophan depletion, GCN2 kinase can be activated, which is related to the proliferation of immune cells [26]. Furthermore, metabolites of the TRP-KYN pathway can act as ligands for the aryl hydrocarbon receptor, the activation of which is able to suppress immune responses [27].

For further investigation, we established a CCl_4_-induced liver fibrosis model in IDO1^–/–^ mice and found that hepatic fibrosis was alleviated compared with the WT model mice. We also discovered that TDO, another rate-limiting enzyme of TRP oxidation, and GCN2 was overexpressed in the liver tissues of the IDO1^–/–^ fibrosis mice compared with the WT fibrosis mice or IDO1^–/–^ control mice. Furthermore, IL-17a and the mRNA expression levels of IL-6, TNF-α and TGF-β1 were decreased in the liver tissues of the IDO1^–/–^ fibrosis mice compared with the WT fibrosis mice.

In contrast to Th1 and Th2 helper T cells, Th17 cells differing from naïve CD4+ T cells were discovered as a new subset of T cells in recent years [28]. Activated Th17 cells can secrete a variety of cytokines, including IL-17a, IL-17f, IL-6, IL-21, and TNF-α. Among these cytokines, IL-17a, mainly produced by Th17 cells, is a characteristic cytokine of Th17 cells and is an important mediator of inflammation [29, 30]. The level of hepatic IL-17a and serum IL-17a were both decreased significantly in the IDO1^–/–^ model mice, indicating that Th17 cells were decreased compared with the WT model mice.

The decreased proliferation of Th17 cells in IDO1^–/–^ fibrosis mice is probably triggered by GCN2 kinase. T cells sense low TRP levels via uncharged tRNAs and subsequently activate GCN2 and initiate an amino acid starvation response, resulting in cell cycle arrest and cell death [31]. The activation of GCN2 can suppress the proliferation of Th17 cells from naïve CD4+ T cells [17]. Thus, the depletion of TRP indirectly induces the number of Th17 cells. The degradation of TRP to KYN is catalysed by the haem dioxygenases IDO1 and TDO [18]. In theory, Th17 cells tend to increase in response to IDO1 deficiency. However, we found that Th17 cells were decreased in the IDO1^–/–^ fibrosis mice. Then, we discovered that the level of TRP in the IDO1^–/–^ fibrosis mice was significantly lower than those in the WT fibrosis mice. Furthermore, the expression of TDO and GCN2 kinase in liver tissues were increased in the IDO1^–/–^ fibrosis mice compared with the WT fibrosis mice. TDO physiologically reduces systemic TRP levels, which are believed to be liver- and neuron specific under normal conditions. Tumours of different origins express TDO, especially melanoma, bladder cancer, hepatocellular carcinoma and glioblastoma [32, 33]. Furthermore, a survey of cancer cell lines indicated that 16% of tumour cell lines are IDO1 positive, while 19% are TDO positive and 15% express both TDO and IDO1 [33]. It has also been reported that systemic TDO inhibition results in increased levels of TRP metabolites, such as KYN, due to the increased availability of TRP for IDO1, as suggested by TDO-deficient mice [34]. However, any changes in the availability and/or level of TDO under the condition of IDO1 deficiency are not yet known. Thus, we hypothesize that under the condition of IDO1 deficiency, TDO increases in the liver to compensate, which can decrease the proliferation of Th17 cells in CCl_4_-induced liver fibrosis. Thus, CD8+ T cells, which can attack injured hepatocytes, are decreased probably in response to the depletion of TRP caused by TDO expression as well.

**Figure 6:**
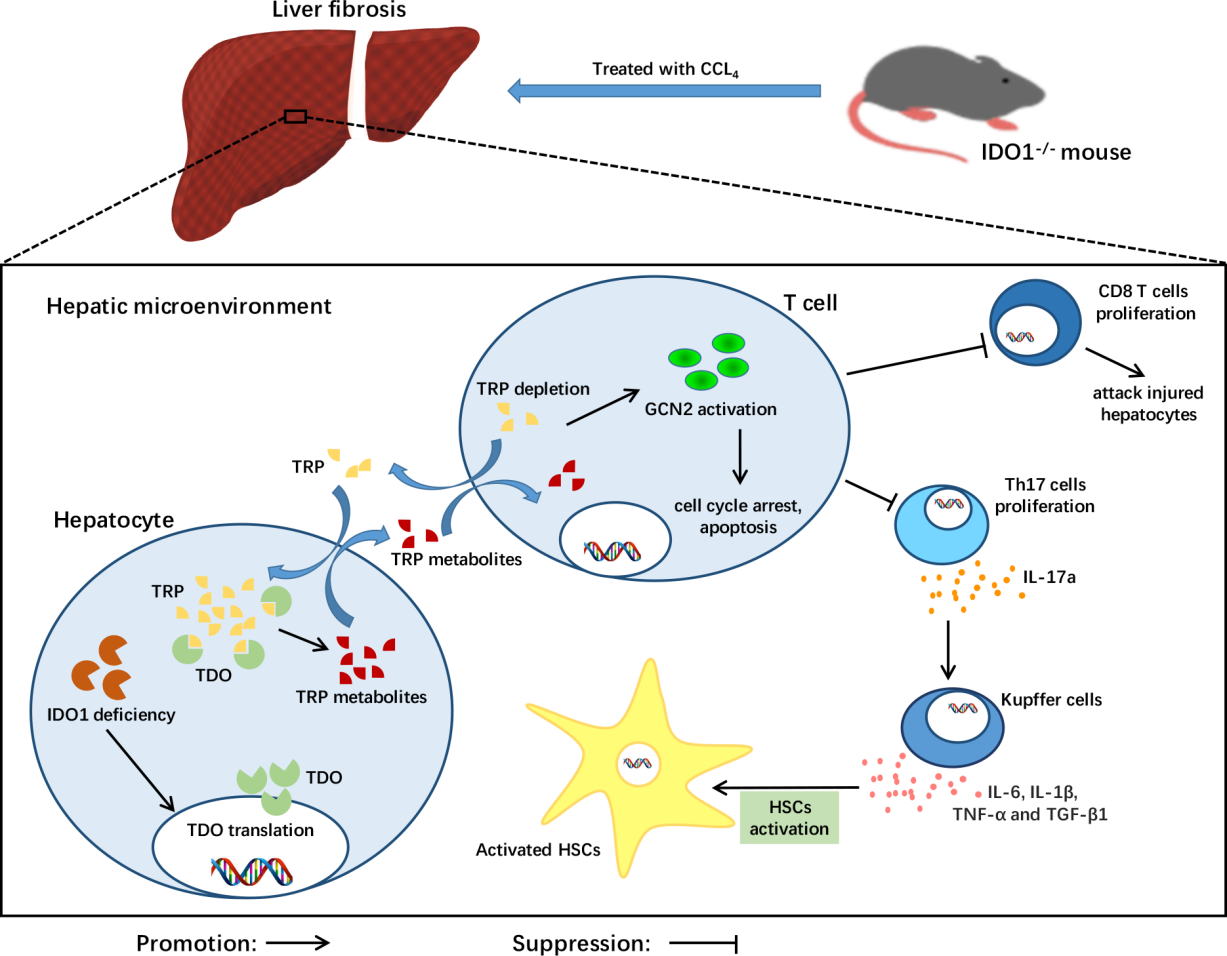
A model depicting the key role of IDO1 deficiency in the pathogenesis of CCl_4_-induced liver fibrosis After treatment with CCl_4_, TDO in liver was increased under the condition of IDO1 deficiency. The compensation of TDO can degrade TRP, the depletion of which activates GCN2 kinase, subsequently suppressing the proliferation of both CD8+ cells and Th17 cells. Liver lesions are ameliorated due to less hepatocyte damage induced by CD8+ cells. HSCs can be activated by cytokines, including IL-6, IL-1β, TNF-α and TGF-β1, that are secreted by Kupffer cells under stimulation with Il-17a. Consequently, IDO1 deficiency protects mice from CCl_4_-induced fibrosis mediated by Th17 cells and TDO compensation.

Recent studies demonstrated that IL-17a was overexpressed in patients with cirrhosis induced by HBV and/or HCV [8, 10]. A similar result was found in CCl_4_-induced mice [35]. Furthermore, IL-17a can promote liver fibrosis. HSCs can be activated by cytokines, including IL-6, IL-1β, TNF-α and TGF-β1, that are secreted by Kupffer cells under stimulation with IL-17a. Moreover, IL-17a can activate HSCs into fibrogenic myofibroblasts via the STAT3 signalling pathway [36]. Consistent with the literature, we found that the expression levels of IL-6, TNF-α and TGF-β1, triggering the activation of HSCs, were decreased significantly in the liver tissues of the IDO1^–/–^ fibrosis mice. This evidence revealed the role of the blockade of Th17 cells in attenuating CCl_4_-induced liver fibrosis in IDO1^–/–^ mice. Thus, we summarized the protective role of IDO1 deficiency in mice during liver fibrosis as showed in Figure 6.

The reason for the positive correlations of the serum IDO1 level with liver lesions and fibrosis degree is not yet known. The up-regulation of IDO1 is intimately linked to several signaling pathways. In this CCl_4_-induced liver fibrosis model, the activation of myeloid dendritic cells induced by cytokines is a promising upstream target [37, 38]. However, a recent study demonstrated that IDO1 deficiency aggravated liver fibrosis in a CCl_4_-induced liver injury model [39]. As liver fibrosis is a dynamic process, IDO1 may play various roles at different stages of hepatic fibrosis. Additionally, changes in TDO in liver tissues, which has been demonstrated to be an alternative route of TRP degradation, were not reported in that study.

In conclusion, we demonstrate that serum IDO1 is a potential hallmark of liver lesions and the degree of liver fibrosis in patients and in an animal model. IDO1 deficiency prevents the progression of hepatic fibrosis induced by CCl_4_, the underlying mechanism of which is related to a decrease in Th17 cells mediated by a compensatory increase in TDO.

## MATERIALS AND METHODS

### Subjects

We enrolled 34 patients with HBV-induced cirrhosis who were followed at the Liver Disease Department of Shenzhen Traditional Chinese Medical Hospital. All the patients were diagnosed by their supervisory doctors and met the definition according to the AASLD Guidelines for Treatment of Chronic Hepatitis B [40]. As controls, we examined 18 HVs at Shenzhen Traditional Chinese Medical Hospital who were negative for HBsAg, anti- HCV antibodies, and human immunodeficiency virus antigen/anti-human immunodeficiency virus antibodies and had no apparent history of liver, autoimmune, or malignant diseases. Both groups were comparable at entry regarding age and male-female ratio. All participants in both groups voluntarily joined this study with informed consents. The study was approved by the ethics committees of Shenzhen Traditional Chinese Medical Hospital and was registered at the Chinese Clinical Trial Registry (ChiCTR-ROC-15007195).

### Animals

All the male IDO^–/–^ mice (strain IDO^tm1Alm/J^) and WT littermates, aged 6–8 weeks, were genotyped according to the Jackson laboratory (Bar Harbor, ME, USA) technical support. A subset of WT mice received daily 1-_D_-MT (Sigma, USA) dissolved in drinking water (5 mg/ml, pH 10.7) [41, 42]. Mice were maintained in pathogen-free facilities at Southern Medical University. All protocols were approved by the Institutional Animal Care and Ethics Committee.

### CCl_4_ model of hepatic fibrosis

The hepatic fibrosis model was described previously [43]. Mice were injected intraperitoneally with 2 μL/g of CCl_4_ (Sigma, USA, adjusted to a 25% concentration in olive oil) or olive oil three times per week for 2 or 4 weeks. The mice were sacrificed after the last dose of CCl_4_, and the livers were removed and processed for further analysis.

### ELISA

Serum samples from human participants and mice were collected at different time points. IDO1 (Cusabio, China), IFN-γ, IL-4 and IL-17a (RayBiotech, USA) immunoassay kits were used according to the manufacturer's instructions. Absorbance at 450 nm was determined using a spectrophotometry analyser (Thermo Fisher Scientific, Finland).

### Biochemical detections

Human serum ALT, AST, GGT, ALB, TB, DB, IB, TBA, CHE and PTA levels were determined using a Cobas ISE 800 Chemistry Analyzer (Roche, Switzerland). Mouse serum ALT and AST levels were detected with an ALT/GPT Assay Kit and AST/GOT Assay Kit (Nanjing Jiancheng Bioengineering Institute, China) according to the manufacturer's instructions. Absorbance at 510 nm was determined using a spectrophotometer (Thermo Fisher Scientific, Finland).

### Histology and immunohistofluorescence

The procedures were following the previous described [44, 45]. For histology, liver tissues from mice were perfused, fixed with 4% paraformaldehyde, penetrated with an ethanol gradient, embedded in paraffin, cut into 3 μm-thick sections and stained with H&E and Sirius red according to standard procedures. For immunohistofluorescence, liver tissues were fixed with 4% paraformaldehyde followed by penetration with a 30% sucrose solution for 24 hours. Livers were sliced into 14 μm-thick sections, which were blocked with blocking buffer containing 0.01 M PBS, 0.1% Triton X-100 and 5% normal goat serum solution for 60 minutes. Subsequently, the sections were separately incubated with primary antibodies, including those against IDO1 (mouse, 1:200, Merck Millipore), α-SMA (rabbit, 1:200, Abcam), CD8a (rat, 1:200, Abcam) and IL-17a (rabbit, 1:50, Affinity Biosciences). The sections were washed and incubated with a secondary antibody, either goat anti-mouse Alexa Fluor 488-conjugated IgG (1:300, Invitrogen) or goat anti-rabbit Alexa Fluor 568-conjugated IgG (1:250, Invitrogen). DAPI (Solarbio Life Science, China) counterstaining was used to stain the nuclei. The sections were coverslipped with fluorescent mounting medium (Dako, Denmark).

### Western blot analysis

Proteins were extracted and dissolved in RIPA cell lysis buffer (Sigma, USA) containing a protease inhibitor cocktail (Sigma, USA) and phosphatase inhibitor cocktail (Sigma, USA). After quantification and denaturation, 30–40 μg protein samples were loaded onto sodium dodecyl sulfate-polyacrylamide gels and transferred to polyvinylidene fluoride membranes (Millipore, Billerica, MA, USA). After blocking, the membranes were incubated overnight at 4°C with primary antibodies, followed by incubation with horseradish peroxidase-conjugated secondary antibodies. Chemiluminescence detection was performed using ECL advance western blotting detection reagents (Millipore, Billerica, MA, USA). Protein expression was detected using antibodies against the following specific antigens: IDO1 (mouse, 1:500, Merck Millipore), α-SMA (rabbit, 1:1000, Cell Signaling Technology), BAX (rabbit, 1:1000, Cell Signaling Technology), cleaved caspase 3 (rabbit, 1:1000, Cell Signaling Technology), TDO (rabbit, 1:1000, Merck Millipore), GCN2 kinase (rabbit, 1:1000, Cell Signaling Technology), IL-17a (rabbit, 1:250, Affinity Biosciences), STAT3 (rabbit, 1:1000, Cell Signaling Technology) and GAPDH (rabbit, 1:2500, Merck Millipore).

### TUNEL assay

The TUNEL assay was performed on frozen liver sections using an *in situ* cell death detection kit, POD (Roche, Switzerland). The detailed protocol outlined in the manufacturer's instructions was followed. The apoptotic index was quantified with image processing software (ImageJ).

### Total RNA isolation, quantitative real-time PCR and genotyping

Reverse transcription of extracted liver total RNA to cDNA was performed using a First Strand cDNA Synthesis kit (Thermo Fisher Scientific). Quantitative real-time PCR was performed using a LIGHTCYCLER^®^ 96 instrument (Roche, Switzerland) with one cycle of 95°C for 10 minutes, followed by 40 cycles of 95°C for 10 seconds, 60°C for 10 seconds and 72°C for 10 seconds using 0.1 μmol/L of gene-specific primers and SYBR Green Supermix (Roche). The primer sequences are listed in Supplementary Table 1A. Genes were normalized to ribosomal RNA, which was the internal control. The genotyping method followed the standard PCR protocols for JAX^®^ Mice, with specific primers for the *IDO1* gene, which are listed in Supplementary Table 1B.

### LC-MS/MS analysis of concentrations of TRP and KYN in liver tissues

Small slices of liver tissues (50 mg) were thawed and then homogenized in ice-cold extraction solution (1:10, w/v). After diluting (1:2, v/v), all the mixtures were transferred to 2mL tubes. After vortex-mixing for 1 minute and centrifugation at 13000 rpm for 15 minute at 4°C, a 100 μL of supernatant was carefully transferred to a 96-well plate. Finally, a 5 μL of supernatant was injected into the LC–MS–MS system (API 3200MD™, AB Sciex Pte.Ltd, USA) for analysis.

### Statistical analysis

All the data are presented as the means ± standard error of the mean (SEM). Statistical differences were evaluated by an unpaired *t* test or one-way analysis of variance, followed by Tukey's multiple comparisons test on dependent experimental designs. Correlations between two groups were assessed using Pearson's coefficient for linear regression. All analyses were performed using GraphPad Prism software (San Diego, CA). Values of *P* < 0.05 were considered statistically significant.

## SUPPLEMENTARY MATERIALS FIGURES AND TABLES



## Abbreviations

HSCs: hepatic stellate cells
ECM: extracellular matrix
Th17: T helper 17
IL-17a: interleukin 17a
IDO: indoleamine 2,3-dioxygenase
TRP: tryptophan
KYN: kynurenine
IFN-γ: interferon-γ
IL-4: interleukin 4
GCN2: general control nonderepressible 2
CCl_4_: carbon tetrachloride
HBV: hepatitis B virus
HVs: healthy volunteers
ALT: alanine transaminase
AST: aspartate transaminase
GGT: γ-glutamyl transferase
ALB: albumin
A/G: albumin/globulin
TB: total bilirubin
DB: direct bilirubin
IB: indirect bilirubin
TBA: total bile acid
CHE: cholinesterase
PTA: prothrombin time activity
α-SMA: α-smooth muscle actin
WT: wide-type
BAX: Bcl-2 associated X protein
TUNEL: terminal deoxynucleotidyl transferase dUTP nick end labeling
1-_D_-MT: 1-Methyl-_D_-tryptophan
TDO: tryptophan 2,3-dioxygenase
IL-6: interleukin-6
IL-1β: interleukin-1β
TNF-α: tumor necrosis factor-α
TGF-β1: transforming growth factor-β 1
STAT3: signal transducer and activator of transcription 3
HCV: hepatitis C virus
SEM: S-nitrosothiol
