# Supplementary material for: Indoleamine 2,3-dioxygenase 1 deficiency attenuates CCl4-induced fibrosis through Th17 cells down-regulation and tryptophan 2,3-dioxygenase compensation
Source: Oncotarget. 2017 Apr 15;8(25):40486–500. doi: 10.18632/oncotarget.17119 (PMC5522192; doi:10.18632/oncotarget.17119)
Supplement: Supplementary file 1 [file oncotarget-08-40486-s001.pdf]

# Indoleamine 2,3-dioxygenase 1 deficiency attenuates $CCl_4$-induced fibrosis through Th17 cells down-regulation and tryptophan 2,3-dioxygenase compensation

## Supplementary Materials

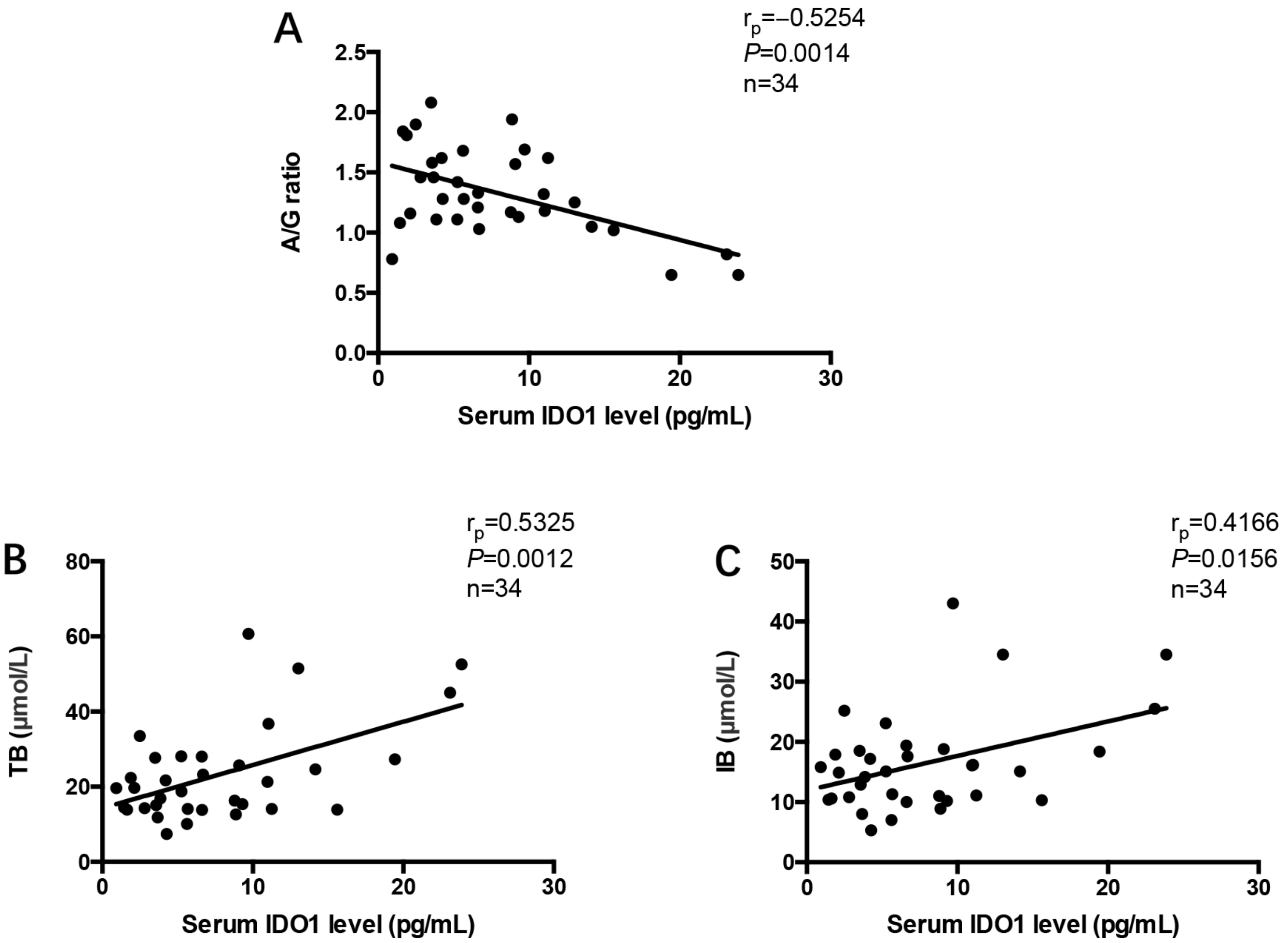


**Supplementary Figure 1:** (**A**), (**B**) and (**C**) Pearson linear correlation tests for IDO1 and A/G ratio, TB and IB levels in the serum of patients. rp: Pearson's correlation coefficient. Data are presented as the mean ± SEM ($^*P < 0.05$, $^{**}P < 0.01$, $^{***}P < 0.001$, $^{****}P < 0.0001$).

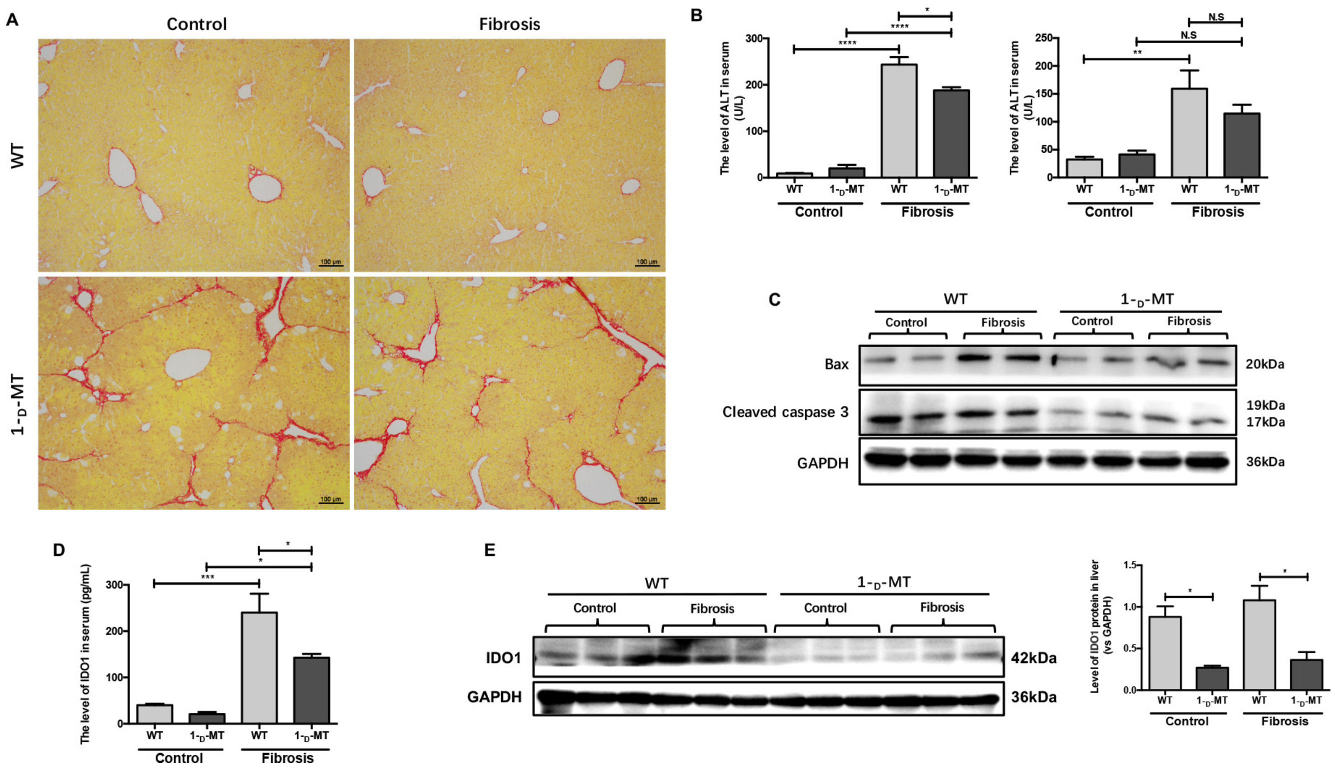


**Supplementary Figure 2:** (**A**) Sirius red staining of liver sections. (**B**) Liver lesions were assessed by measuring serum ALT and AST levels in WT and 1-D-MT mice. (**C**) Western blot analysis of the expression of α-SMA, Bax and cleaved caspase 3 in WT and 1-D-MT mice. (**D**) ELISA evaluating the serum IDO1 level in WT and 1-D-MT mice. (**E**) Western blot analysis of the expression of IDO1 in WT and 1-D-MT mice. The data are presented as the means ± SEM (**P* < 0.05, ***P* < 0.01, ****P* < 0.001, *****P* < 0.0001).

## Supplementary Table 1A: Primer sequences for quantitative real-time PCR

| Target gene | Forward primer (5′→3′) | Reverse primer (5′→3′) |
|---|---|---|
| IL-17a | GCTGACCCCTAAGAAACCCC | GAAGCAGTTTGGGACCCCTT |
| IL-6 | CCCCAATTTCCAATGCTCTCC | CGCACTAGGTTTGCCGAGTA |
| TGF- β1 | CAACCCAGGTCCTTCCTAAA | GGAGAGCCCTGGATACCAAC |
| TNF-α | AGGGTCTGGGCCATAGAACT | CCACCACGCTCTTCTGTCTAC |
| IL-1β | GGTCAAAGGTTTGGAAGCAG | TGTGAAATGCCACCTTTTGA |

## Supplementary Table 1B: Primer sequences for mice genotyping

| Primer | 5′ Label | Sequence 5′→3′ | 3′ Label | Label Primer Type |
|---|---|---|---|---|
| oIMR4164 | | TGG AGC TGC CCG ACG C | | Wild type Forward |
| oIMR6916 | | CTT GGG TGG AGA GGC TAT TC | | Mutant Forward |
